# Necrostatin-1 Attenuates Ischemia Injury Induced Cell Death in Rat Tubular Cell Line NRK-52E through Decreased Drp1 Expression

**DOI:** 10.3390/ijms141224742

**Published:** 2013-12-18

**Authors:** Li Zhang, Fen Jiang, Yuanhan Chen, Jialun Luo, Shuangxin Liu, Bin Zhang, Zhiming Ye, Wenjian Wang, Xinling Liang, Wei Shi

**Affiliations:** 1 Southern Medical University, Guangzhou 510080, China; E-Mail: zhanglichangde@163.com; 2 Department of Nephrology, Guangdong General Hospital, Guangdong Academy of Medical Sciences, 106 Zhongshan No. 2 Road, Guangzhou 510080, China; E-Mails: gdmc99@163.com (Y.C.); allanlaw@126.com (J.L.); liushuangxii@163.com (S.L.); binzhang52028@163.com (B.Z.); yezming@163.com (Z.Y.); yezming@163.com (W.W.); 3 Department of Nephrology, the First Affiliated Hospital of Nanhua University, Hengyang 421001, China; E-Mail: jiangfengyes@163.com

**Keywords:** Necrostatin-1, cell death, renal ischemia injury, dynamin-related protein 1

## Abstract

Necrostatin-1 (Nec-1) inhibits necroptosis and is usually regarded as having no effect on other cell deaths. Here, this study explored whether the addition of Nec-1 has an effect on cell death induced by simulated ischemia injury in rat tubular cell line NRK-52E. In addition, we also investigated the mechanism of Nec-1 attenuates cell death in this renal ischemia model. The NRK-52E cells were incubated with TNF-α + antimycinA (TA) for 24 h with or without Nec-1. Cell death was observed under fluorescent microscope and quantified by flow cytometry. Cell viabilities were detected by MTT assay. The protein expression of dynamin-related protein 1 (Drp1) was detected by Western blotting and immunofluorescence assay. Increased cell death in simulated ischemia injury of NRK-52E cells were markedly attenuated in the Nec-1 pretreated ischemia injury group. Meanwhile, cell viability was significantly improved after using Nec-1. In addition, we also observed that the protein expression of Drp1, a mediator of mitochondrial fission, was significantly increased in simulated ischemia injury group. Increased Drp1 expression in the ischemia injury group can be abolished by Nec-1 or Drp1-knock down, accompanied with decreased cell death and improved cell viabilities. These results suggest that Nec-1 may inhibit cell death induced by simulated ischemia injury in the rat tubular cell line NRK-52E through decreased Drp1 expression.

## 1. Introduction

Acute kidney injury (AKI) induced by renal ischemia leads to significant morbidity and mortality [1]. A number of pathologic processes contribute to AKI, including endothelial and epithelial cell death, intratubular obstruction, changes in local microvascular blood flow, as well as immunological and inflammatory processes [2]. The relative contribution of each to AKI is uncertain. Limited understanding of the cellular mechanisms of AKI complicates the development of an effective treatment. Recently, there are many *in vivo* and *in vitro* studies supporting a pathogenic role for different forms of cell death, including necrosis, apoptosis, and autophagy in AKI [2–5]. This indicates that targeting these cell deaths will probably improve the outcome of AKI.

Necroptosis is a recently discovered, regulated form of programmed necrosis initiated by the activation of tumor necrosis factor alpha (TNF-α) and/or Fas that is distinct from caspase-dependent apoptotic cell death [6]. Necrostatin-1 (Nec-1), a small molecule inhibitor, originally identified by Degterev in a chemical library screen, was found to selectively target the kinase activity of RIP1, a key mediator of necroptosis [7,8]. Nec-1 is commercially available and has been used extensively both *in vitro* and *in vivo* by multiple groups to elucidate the role of necroptosis [9–13]. Usually, it does not protect against caspase-dependent apoptosis, or against other programmed cell death, such as autophagy [7]. However, subsequent studies showed that Nec-1 had an effect on autophagy and apoptosis in some cell injury models [14,15]. A recent study also demonstrated that Nec-1 prevented osmotic nephrosis and contrast-induced AKI in Mice [16]. Given the aforementioned roles of Nec-1, we then asked whether Nec-1 affects the cell injury of AKI induced by simulated ischemia in the rat tubular cell line NRK-52E.

In the present study, we explored whether the addition of Nec-1 had a protective effect on cell injury, induced by simulated ischemia, in rat tubular cell line NRK-52E, In addition, we also investigated the mechanism of Nec-1 that attenuates cell injury in this renal ischemia model.

## 2. Results and Discussion

### 2.1. Results

#### 2.1.1. Nec-1 Inhibits Cell Death Induced by Simulated Ischemia Injury in Rat Tubular Cell Line NRK-52E with TNF-α Stimulation and ATP Depletion

**Figure 1.**
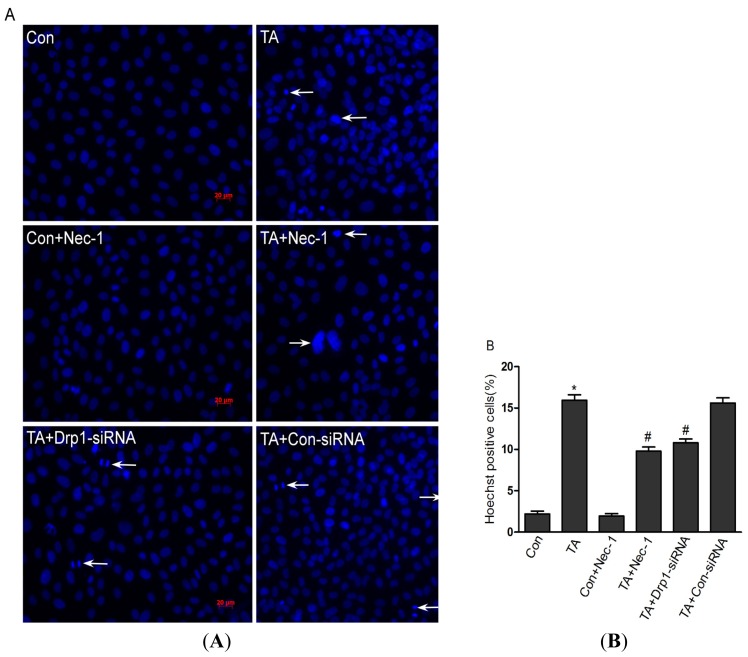
Nec-1 protected against simulated ischemia-induced cell death measured by Hoechst 33258 staining. (**A**) NRK-52E cells were exposed to simulated ischemia injury of NRK-52E cells with TNF-α stimulation and ATP depletion. After that, NRK-52E cells were incubated with Nec-1 (20 μM) or *Drp1*-siRNA (50 nM). Then, cells were stained with Hoechst 33258 (blue) Images were obtained with a fluorescent microscope. All the nuclei are uniformly weak-stained in the control group. White arrows indicate the nuclei of cell deaths (bright blue). Original magnification: ×200; (**B**) Quantification of Hoechst positive cells (%) in NRK-52e cells treated with different treatments. Data were counted from 200 cells per field and twenty fields were analyzed in each group. Note: *p < 0.01, *vs.* Con group; # *p* < 0.01, *vs.* TA group.

**Figure 2.**
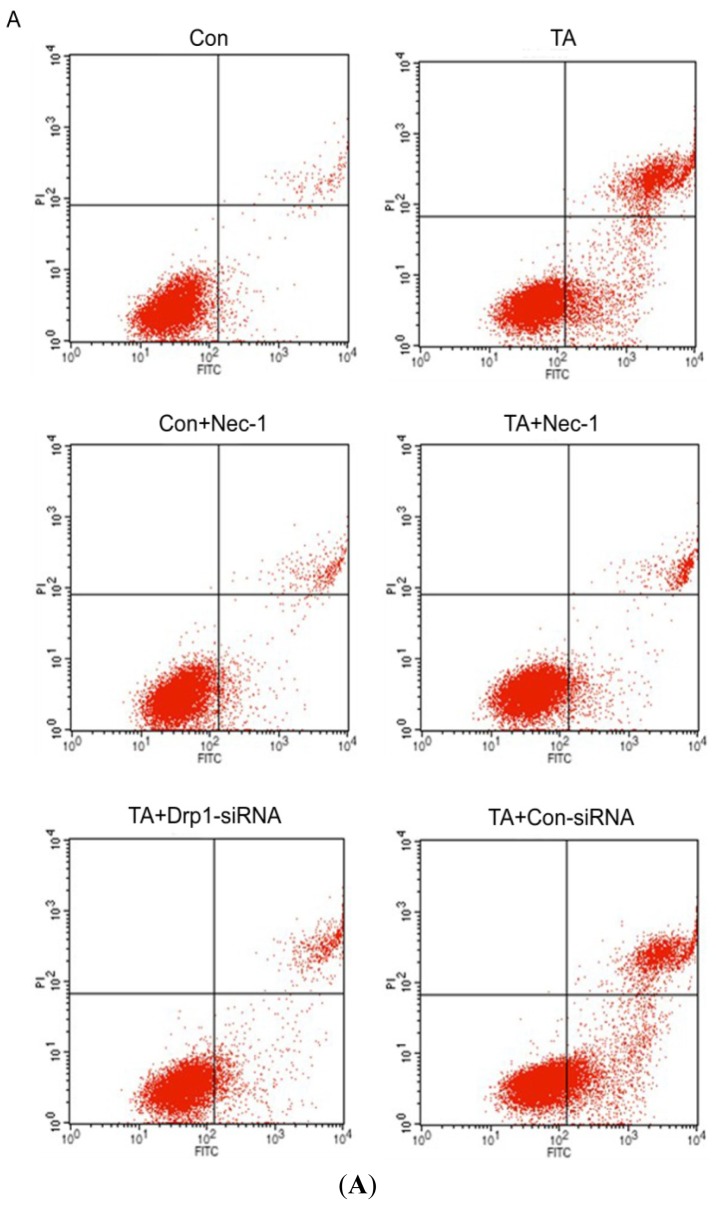

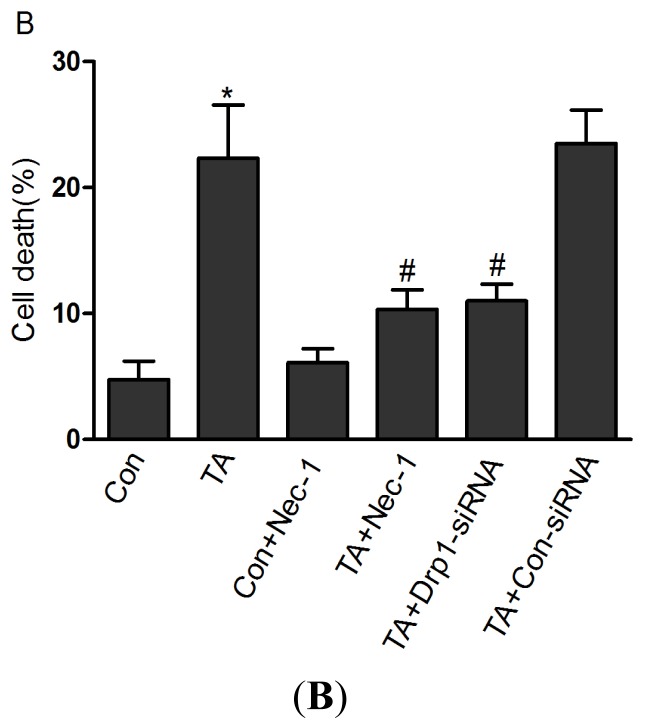
Effect of Nec-1 and *Drp1*-siRNA on cell death of NRK-52e cells induced by ischemia injury determined by flow cytometry using Annexin-FITC/PI staining. (**A**) Flow cytometry displayed the effect of Nec-1 on cell death of NRK-52E cells induced by ischemia injury with TNF-α stimulation and ATP depletion; (**B**) Quantification of cell death percentages of the NRK-52E cells with different treatments. Note: *p < 0.01, *vs.* Con group; # *p* < 0.05, *vs.* TA group.

To evaluate the effect of Nec-1 on ischemia injury induced cell death, cells were subsequently stained with Hoechst and Annexin-FITC/PI, and visualized by fluorescence microscopy. Cell death stained with Hoechst appears as an intense blue fluorescence. As shown in Figure 1, the number of cell deaths was significantly increased in the TA group, however, when these cells were Nec-1 pretreated, the number of cell deaths was markedly decreased. Similarly, a significant increase in the percentage of cell deaths was observed using flow cytometry between the control group and the TA group, 4.77% ± 1.48% and 22.33% ± 4.24%, respectively (*p* < 0.01) (Figure 2). The Nec-1 pretreatment protects cells from cell death caused by ischemia injury. The Nec-1+TA group showed 10.34% ± 1.55% cell death, while the TA group showed 22.33% ± 4.24% (*p* < 0.05) (Figure 2). The use of inhibitor Nec-1 (20 μM) alone had no effect on cell death (4.77% ± 1.48% and 6.11% ± 1.10%, respectively) (*p* > 0.05).

#### 2.1.2. Nec-1 Increased Cell Viability in Rat Tubular Cell Line NRK-52E after TNF-α Stimulation and ATP Depletion

**Figure 3.**
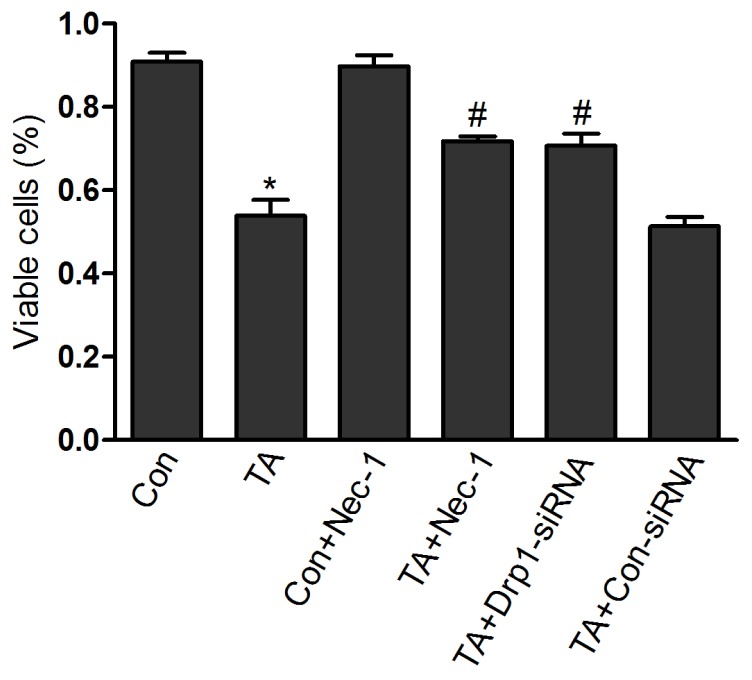
Cell viability was measured by the MTT assay and expressed as percentages of control. Data are mean ± SEM from six independent experiments. Note: *p < 0.05, *vs.* Con group; # *p* < 0.05, *vs.* TA group.

As shown in Figure 3, cell viability was significantly decreased in the TA group (53.88% ± 3.82%), compared to control group (90.87% ± 2.20%) (*p* < 0.01). After treatment with Nec-1 (20 μM) for 24 h, we observed that Nec-1 pretreatment markedly increased cell viability from 53.88% ± 3.82% (TA group) to 71.75% ± 1.21% (Nec-1 + TA group) (*p* < 0.01). The pretreatment with Nec-1 (20 μM) had no effect on cell viability in the control group. We can see this by comparing the Nec-1 + control group (89.78% ± 2.64%) with the control group (90.87% ± 2.20%) (*p* > 0.05).

#### 2.1.3. Nec-1 Inhibits Increased Drp1 Protein Expression in Rat Tubular Cell Line NRK-52E after TNF-α Stimulation and ATP Depletion

**Figure 4.**
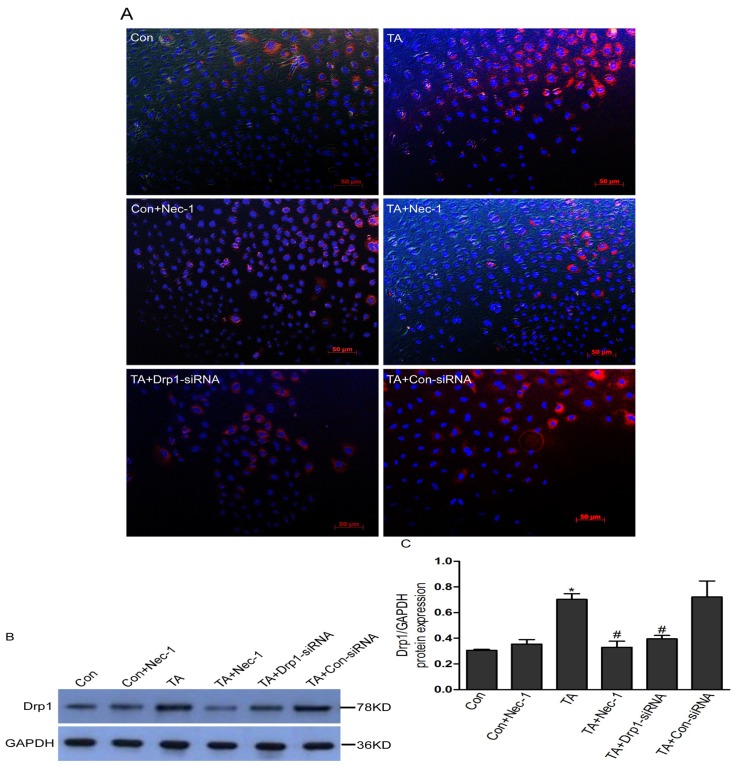
Effects of Nec-1 and *Drp1* gene knock down on the protein expression of Drp1 in NRK-52E cells treated with TNF-α stimulation and ATP depletion. (**A**) Confocal images of NRK-52E cells showing expression of Drp1 (red) and DAPI-stained nuclei (blue); (**B**) Nec-1 treatment decreased the expression of Drp1 detected by immunoblot in an extract of stimulated ischemia injury of NRK-52E cells with TNF-α stimulation and ATP depletion; (**C**) Densitometric analysis of three repetitions of the experiment shown in (**C**). Values are expressed as the mean ± SEM. *p < 0.01 *vs.* Con; # *p* < 0.01 *vs.* TA. Ischemia injury increased the protein expression of Drp1 in NRK-52E cells, and the effect was blocked by either Nec-1, a specific inhibitor of necroptosis, or *Drp1*-siRNA.

To explore the underlying mechanism of Nec-1 action, we studied the protein expression of Drp1 in NRK-52E cells under ischemia injury condition. Figure 4 demonstrates the protein expression of Drp1 using immunofluorescent staining. As shown in Figure 4, the Drp1 protein expression was markedly increased in ischemia injury NRK-52E cells (TA group). However, the increased trend was significantly blocked after using Nec-1. Similarly, immunoblotting, also exhibited the increased Drp1 protein, was significantly inhibited with the addition of Nec1 (Figure 4), which is consistent with the notion that Nec-1 inhibits Drp1 protein expression in rat tubular cell line NRK-52E suffering from ischemia injury.

#### 2.1.4. Drp1 Mediates Ischemia Injury-Induced Cell Death in Rat Tubular Cell Line NRK-52E with TNF-α Stimulation and ATP Depletion

To further study the role of Drp1 in ischemia injury of NRK-52E cells, we employed *Drp1* knock down, to assess the effect of Drp1 on NRK-52E cell injury resulting from TNF-α stimulation and ATP depletion. As shown in Figure 2, we observed that the cell death-inducing effect of ischemia injury was abolished by pretreatment with *Drp1* knock down (22.33% + 4.24% *vs.* 11.01% + 1.33% (Figure 3)). Meanwhile, we also observed that cell viability was significantly improved in the Nec-1 + TA group (70.73% ± 2.86%), compared to TA control group (53.88% ± 3.82%) (*p* < 0.01, Figure 3). These results suggest that Drp1 mediates ischemia injury-induced cell death.

### 2.2. Discussion

AKI induced by renal ischemia is a common clinical complication characterized by an abrupt decrease in the glomerular filtration rate (GFR). Despite supportive care, including renal replacement therapy, the five-year mortality after AKI remains ~50% [17]. Limited understanding of the cellular mechanisms of AKI induced by renal ischemia complicates the development of an effective treatment. Abundant evidence shows that necrosis, apoptosis, necroptosis, and autophagy are involved in AKI [2–5]. This indicates that targeting these cell deaths will probably improve the outcome of AKI.

Emerging evidences suggest that necroptosis is closely related to other cell death, at least in particular conditions [14,18,19]. Recently, Linkermann *et al.* also demonstrated the pathophysiological coexistence and corelevance of cyclophilin (Cyp)D-mediated mitochondrial permeability transition (MPT) and receptor interacting protein kinase (RIPK)1-mediated necroptosis [20]. The parallel existence of the two separate pathways that induce regulated necrosis in the same organ, following the same ischemic stimulus, demonstrates the complexity in the pathophysiology of ischemia-reperfusion injury. Nec-1 is a key mediator of necroptosis, originally identified, selectively targets the kinase activity of RIP1. It has recently been discussed to directly influence other cell deaths, or to have other functions, in addition to its effects to block necroptosis [14,18,19,21]. Thus, further understanding of these additional roles of Nec-1 promise new therapeutic possibilities.

In the present study, we hypothesized that administration of Nec-1 could produce an effect on ischemia-induced renal tubular epithelial cell death. To test our hypothesis, we simulated ischemic AKI in rat tubular cell line NRK-52E with TNF-α stimulation and ATP depletion, and then we examined the potential effects of Nec-1 on ischemia-induced cell death. In addition, we also explored the potential mechanism of Nec-1 on cell death in, such a renal ischemia model.

In this study, we found that cell death, determined by Hoechst staining and flow cytometry with annexin V and PI double stains, was significantly increased in simulated ischemia injury of NRK-52E cells with TNF-α stimulation and ATP depletion. However, the effect of ischemia injury induced cell death in NRK-52E cells was blocked by concomitant treatment with Nec-1. Similarly, we also found cell viability was markedly improved in ischemia injury of NRK-52E cells after using Nec-1. These results suggest that Nec-1 has a protective effect on ischemia injury of renal tubular epithelial cells.

To further explore the mechanisms of Nec-1 inhibited ischemia injury-induced cell death, we next investigated the protein expression of Drp1, a member of the dynamin family of large GTPases [22,23]. Drp1 is a primarily cytoplasmic protein which, when activated, can form ring-like multimers and translocate to the mitochondria. There, in concert with accessory proteins, Drp1 facilitates mitochondrial division [23]. A body of evidence has accumulated to suggest that Drp1 contributes to cell death [24–26]. Drp1 foci accumulate on mitochondria and mediate dramatic mitochondrial fission prior to caspase activation. Inhibiting Drp1 activity delays cytochrome C release, caspase activation, and subsequent steps in cell demolition [27]. In this study, the protein expression level of Drp1 in cultured NRK-52E cells was significantly increased under simulated ischemia injury with TNF-α stimulation and ATP depletion, whereas Nec-1 or *Drp1*-knock down treatment strongly abolished the elevation of Drp1 level induced by ischemia injury. Meanwhile, we also found that the cell injury induced by ischemia injury in NRK-52E cells was markedly improved after *Drp1* knock down. These results indicated that Nec-1 protects renal tubular epithelial cell from ischemia injury, probably through a mechanism dependent on Drp1.

This new finding will support a notion that Nec-1, which was originally identified to selectively target the kinase activity of RIP1, can also mediate cell death through Drp1, a member of the dynamin family of GTPases required for mitochondrial fission. However, one fact we must admit is that, in the present study, we cannot rule out the probability of the presence of other molecular mechanisms on Nec-1 playing such a protective effect in this ischemia injury model. Especially, one recent study from Mohib *et al.* demonstrated that Indoleamine 2,3-dioxygenase (IDO), which is the rate-limiting enzyme in the kynurenine enzymatic pathway, is overexpressed in tubular epithelial cells subject to ischemia reperfusion injury, and IDO could promote ischemia-reperfusion injury by augmenting tubular cell death and worsening function [28]. Therefore, whether the presence of the inhibitory activity of Nec-1 against IDO is still unknown, and this will need to be clarified in further studies.

## 3. Experimental Section

### 3.1. Reagents

Recombinant human TNF-α was obtained from PeproTech Inc. (Suzhou, China) Necrostatin-1 (Nec-1) and antimycin A were obtained from Enzo Life Sciences Inc. (Shanghai, China). Rabbit Drp1 antibody (#8570) monoclonal antibody was obtained from Cell Signaling Technology, Inc. (Shanghai, China). GAPDH antibody (BP4233) and Goat anti-Rabbit IgG-HRP labeled secondary antibody (BA1055) were obtained from Boster Technology, Inc. (Wuhan, China). Goat anti-rabbit IgG-PE (sc-3739) was obtained from Santa Cruz Biotechnology, Inc. (Dallas, TX, USA). The small interfering RNA (siRNA) sequences that target *Drp1* (sc-270298) and control siRNA (sc-37007) were obtained from Santa Cruz Biotechnology, Inc. (Dallas, TX, USA).

### 3.2. Cell Cultures

NRK-52E cell stocks (a renal tubule epithelium cell line from normal rat) were obtained from American Type Culture Collection (CRL-1571; Manassas, VA, USA). Cells were grown in 25-cm^2^ cant-necked, vent-cap, uncoated flasks (Becton Dickinson, Oxnard, CA, USA), and propagated in RPMI-1640 medium supplemented with 10% heat-inactivated fetal bovine serum (FBS) (Gibco) plus 100 U mL^−1^ penicillin and 100 μg mL^−1^ streptomycin (Sigma, Shanghai, China) in an atmosphere of 5% CO_2_ in air at 37 °C. Medium was replaced every three days. Cells were subcultured when the cell monolayer reached 80% confluence. Then cells were exposed to different experimental conditions.

### 3.3. ATP Depletion

ATP depletion, an established model of renal ischemia, is induced by exposing cells to prewarmed glucose-free DMEM that contained the electron transport chain inhibitor antimycin A (10 μM) at 37 °C in the presence of 5% CO_2_, following previously described protocols [29,30]. Glucose removal was required in order to prevent cellular anaerobic glycolysis. Cells were subjected to 24 h of energy deprivation.

### 3.4. Cell Treatment

Cells were divided into six groups as follows: (1) Control group: cells were maintained in RPMI-1640 medium supplemented with 10% FBS for 24 h; (2) TA group: cells were treated with 10 ng/mL of TNF-α and 10 μM antimycin A for 24 h; (3) Nec-1 + control group: cells were pretreated with 20 μM of Nec-1 for 24 h; (4) Nec-1 + TA group: cells were pretreated with 20 μM of Nec-1, 10 ng/mL of TNF-α and 10 μM antimycin A for 24 h; (5) TA + *Drp1*-siRNA group: cells were pretreated with 50 nM of Drp1-siRNA for 24 h, and then continuously treated with 10 ng/mL of TNF-α and 10 μM antimycin A for 24 h; and (6) TA + Con-siRNA group: cells were pretreated with 50 nM of scrambled siRNA for 24 h, and then continuously treated with 10 ng/mL of TNF-α and 10 μM antimycin A for 24 h. siRNA knock down was performed according to the manufacturer’s instructions.

### 3.5. Annexin V and Propidium Iodide Stains

AnnexinV and propidium iodide (PI) stains were performed by using AnnexinV-FITC Apoptosis Detection Kit (Nanjing KeyGEN Biotech. Co. Ltd., Nanjing, China) according to manufacturer’s protocol. Briefly, the cell pellet was resuspended in 1× binding buffer followed by incubation with 5 μL of Annexin V (conjugated with FITC) and 5 μL of PI in the dark for 10 min. Cell fluorescence was then analyzed using a Cell Lab Quanta^™^ SC Flow cytometer (Beckman Coulter, Inc, Fullerton, CA, USA). Cells positive for Annexin V-FITC and negative for PI were considered cell death. All experiments were repeated three times.

### 3.6. Hoechst 33258 Stains

NRK-52E cells were seeded on sterile cover glasses placed in the six-well plates at a density of 1.0 × 10^4^ cells cm^−2^ and cultured at 37 °C under different experimental treatments. Subsequently, cells were fixed, washed twice with PBS, and stained with Hoechst 33258 staining solution according to the manufacturer’s instructions (Beyotime, Jiangsu, China). Stained nuclei were observed under a confocal microscope (LCSM, Zeiss KS 400, Postfach, Germany).

### 3.7. Cell Viability Assay

NRK-52E cells under different experimental conditions were harvested for MTT assays (Nanjing KeyGEN Biotech. Co. Ltd., Nanjing, China) to determine the viable cell numbers, according to the manufacturer’s instructions. Briefly, approximately 5 × 10^3^ cells were plated onto each well of a 96-well plate for 24 h, followed by different treatment as mentioned above. After incubation, 20 μL of MTT dye solution (5 mg/mL in PBS, pH 7.4) was added to wells containing 180 μL of growth medium. After 4 h of incubation at 37 °C, the medium was replaced with 200 μL of DMSO. After gently shaking at 37 °C for 10 min, MTT reduction was quantified spectrophotometrically at 570 nm using an Infinite M200 microplate reader. The assay was repeated six times. The cell viability was calculated as following: cell viability (%) = OD (experiment) − OD (blank)/OD (control) − OD (blank) × 100%.

### 3.8. Immunofluorescence

After subjected to various treatments, NRK-52E cells were fixed with 4% paraformaldehyde at −20 °C for 20 min, and then were blocked with 5% BSA for 20 min at room temperature (RT) to block nonspecific binding. Then, the cells were incubated with Drp1 antibody (Cell Signaling Technology, Shanghai, China; 1:50) overnight at 4 °C. After three washes with PBS, cells were incubated with the goat anti rabbit IgG-PE (Santa Cruz, Dallas, TX, USA; 1:100) for 1 h at RT, and then double stained with 4′-6-diamidino-2-phenylindole (DAPI) for 5 min to visualize the nuclei. After washing, the slides were mounted with antifade mounting medium (Beyotime, Jiangsu, China). Photomicrographs were taken with confocal microscopy (LCSM, Zeiss KS 400, Postfach, Germany). All images were analyzed by two investigators blinded to the identity of the samples.

### 3.9. Western Blotting

Cells under different experimental conditions were lysed with RIPA lysis buffer. The samples were centrifuged and the supernatants were collected as total cell extracts. Protein concentration was determined by the bicinchoninic acid (BCA) protein assay (Beyotime, Jiangsu, China). An aliquot of cell lysates containing 40 μg of protein were separated by sodium dodecyl sulfate (SDS)/polyacrylamide gel electrophoresis, and transferred to a polyvinylidene difluoride membrane (Immobilon-P, Millipore, MA, USA) by electroblotting. After blocking, the membranes then were incubated overnight at 4 °C with rabbit monoclonal antibody to Drp1. After washing, the secondary antibody was added and incubated 1 h at RT. Protein bands were visualized by ECL Plus Western Blotting Detection Reagents (Pluslight, Forevergen, Shanghai, China), and then exposed to X-ray film (Kodak, Rochester, NY, USA). The bands of the resulting autoradiographs were quantified densitometrically using Bandscan software (Version 5.0; Funglyn Biotech, Toronto, ON, Canada). Protein expression was quantified as the ratio of specific band to GAPDH.

### 3.10. Statistical Analysis

All values are expressed as mean ± SEM. Statistical analysis was performed using the statistical package SPSS for Windows Ver. 17.0 (SPSS, Inc., Chicago, IL, USA). Multiple comparisons among the groups were conducted by one-way analysis of variance with Turkey’s tests for *post hoc* analysis. *p*-values < 0.05 were considered significant.

## 4. Conclusions

Taken together, here, for the first time, we investigated the potential effects of Nec-1 on ischemia-injury *in vitro* cultured NRK-52E cells with TNF-α stimulation and ATP depletion. We found that Nec-1, identified as a specific inhibitor of RIP1, can effectively attenuate cell death induced by simulated ischemia injury in NRK-52E cells. In addition, we also demonstrated Nec-1 protect renal tubular epithelial cell from ischemia injury-induced cell death probably through decreased the expression of Drp1.
